# Modulation of Cytokine Release and Gene Expression by the Immunosuppressive Domain of gp41 of HIV-1

**DOI:** 10.1371/journal.pone.0055199

**Published:** 2013-01-30

**Authors:** Joachim Denner, Magdalena Eschricht, Michael Lauck, Marwan Semaan, Philipp Schlaermann, Hyunmi Ryu, Levent Akyüz

**Affiliations:** Robert Koch Institute, Berlin, Germany; Rush University, United States of America

## Abstract

The transmembrane envelope protein gp41 of the human immunodeficiency virus HIV-1 plays an important role during infection allowing fusion of the viral and cellular membrane. In addition, there is increasing evidence that gp41 may contribute to the immunodeficiency induced by HIV-1. Recombinant gp41 and a synthetic peptide corresponding to a highly conserved domain in gp41, the immunosuppressive (isu) domain, have been shown to inhibit mitogen-induced activation of human peripheral blood mononuclear cells (PBMCs) and to increase release of IL-6 and IL-10 from these cells. We recently reported that a single mutation in the isu domain of gp41 abrogated the immunosuppressive properties and that HIV-1 sequences containing such abrogating mutations had never been isolated from infected individuals. Here, we studied the influence of the isu peptide on the release of 66 cytokines and the expression of 27,000 genes in PBMCs. Incubation of PBMCs with isu peptide homopolymers increased the expression of 16 cytokines among them IL-6 and IL-10, and decreased that of IL-2 and CXCL9. Interestingly, the extend of cytokine modulation was donor-dependent. Among the genes up-regulated were IL-6, IL-8, IL-10 but also MMP-1, TREM-1 and IL-1beta. Most importantly, genes involved in innate immunity such as FCN1 and SEPP1 were found down-regulated. Many changes in cytokine expression demonstrated in our experiments were also found in HIV-1 infected individuals. These data indicate that the isu domain of gp41 has a broad impact on gene expression and cytokine release and therefore may be involved in HIV-1 induced immunopathogenesis.

## Introduction

**Figure 1:**
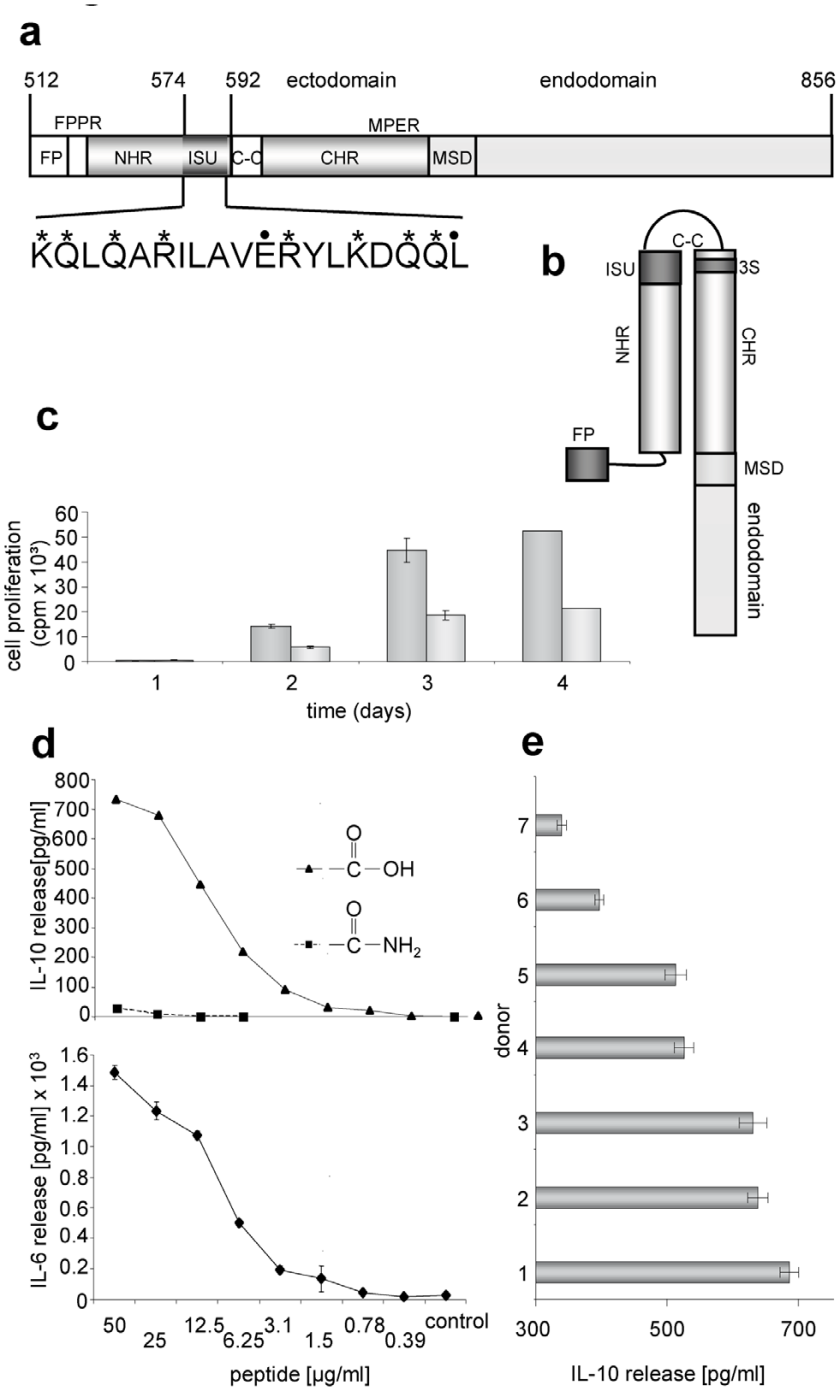
Localisation and activity of the immunosuppressive (isu) domain of gp41 of HIV-1. (a) Functional domains in gp41 of HIV-1 (accession-nr. NCBI K03455): FP, fusion peptide; FPPR, fusion peptide proximal region; NHR, N-terminal helical region; ISU, isu domain; S-S, cystein-cystein loop; CHR, C-terminal helical region; MPER, membrane proximal external region; MSD, membrane spanning domain, 3S, domain binding to the gC1qR and inducing NKp44L expression. In the amino acid sequence of the isu domain stars (*) indicate NH_2_-groups, points (.) mark COOH groups relevant for polymerisation. (b) Localisation of the isu domain after interaction of the NHR with the CHR generating a six helix bundle (only one molecule of the trimer is shown). (c) Influence of the isu-peptide on the proliferation of PHA stimulated PBMCs from a healthy donor. Cell proliferation was measured by ^3^H-thymidine incorporation. ^3^H-thymidine was added on day zero, one, two or three and cells were then harvested one the next day and the counts per minute were determined, gray - medium control, dark gray – isu peptide-BSA conjugates, added at day 0. (d) Dose dependent induction of IL-6 and Il-10 release by the isu peptide homopolymer (triangle) as measured in ELISAs. In contrast, the amidated control peptide (square) is inactive. (e) Comparative ELISA analysis of IL-10 release from PBMCs of seven donors all treated with the same amount and batch of the isu-peptide homopolymer, the IL-10 release of their PBMCs incubated with medium alone was zero. The p values were calculated using the Student's t-test, n = 3. The p value for the high responder donor 1 was p = 0.001, that of the low responder donor 7 p = 0.03.

The transmembrane envelope (TM) protein gp41 of the human immunodeficiency virus type 1 (HIV-1) facilitates - like the TM proteins of all retroviruses - the fusion of the viral and the cellular membranes during infection [1]. In addition to this function a contribution of TM proteins to the induction of the immunodeficiency was proposed on the basis of numerous findings: First, all retroviruses including HIV-1 and HIV-2 are immunosuppressive when present at a critical viral load in the infected host. This was studied in detail for gammaretroviruses such as the murine leukaemia virus (MuLV) and the feline leukaemia virus (FeLV). During these investigations it became clear that non-infectious virus particles and the corresponding TM proteins were immunosuppressive in different *in vitro* and *in vivo* assays (for review see [2], [3]), indicating that the TM proteins may contribute to immunosuppression. Second, transfection and expression of different retroviral TM proteins on the surface of tumour cells growing to tumours in immunocompromised, but not in immunocompetent mice, made these cells to grow in the immunocompetent animals, thus demonstrating the immunosuppressive activity of the retroviral TM proteins *in vivo* [4], [5]. Third, synthetic peptides corresponding to a domain of the TM proteins localised in the C-terminal part of the N-helical repeat, the so-called immunosuppressive (isu) domain (Figure 1a), inhibited the activation of mitogen-triggered PBMCs [6]–[10]. The isu domain is highly conserved among retroviruses [9] including different strains of HIV-1, HIV-2 and the simian immunodeficiency viruses (SIV) (Supplementary figure S1). Interestingly, the isu domain is located opposite the 3S domain when gp41 is present in a so-called six helix bundle conformation allowing interaction of the C-terminal and N-terminal helical regions of gp41 (Figure 1b). The 3S region was shown to bind to the receptor for the globular domain of C1q (gC1qR), to induce NKp44L expression on CD4^+^ cells (an activator ligand of the natural cytotoxicity receptor NKp44) and it is thought to contribute to the decline of the number of CD4^+^ cells [11]. Synthetic peptides (17- to 19-mers) corresponding to the isu domain of gammaretroviruses and HIV-1 were biologically active only when conjugated to a carrier protein. They modulated the cytokine release of PBMCs from healthy donors, for example, they caused an increase of IL-10 and they had an inhibitory effect on protein kinase C (PKC) [12]–[16]. Fourth, recombinant gp41 modulated cytokine expression of normal human PBMCs in the same manner [17]–[19], suggesting that the isu domain of gp41 may contribute to the immunodeficiency induced by HIV-1 [20]. However, since this gp41 was produced in *E.coli*, a contamination with endotoxin also inducing IL-10 could not be excluded.

In parallel we showed that recombinant gp41 of HIV-1 produced in human 293T cells that is free of endotoxin, that is glycosylated and in a trimeric conformation also modulated expression of IL-10, IL-6 and other genes in the same manner as the synthetic isu peptide [21]. Single mutations in the isu domain abrogated the ability to cause IL-10 release and to modulate gene expression. Replication competent virus particles with such mutations in gp41 did not induce IL-10 release, whereas the wild-type virus did. Since identical changes in cytokine release were seen upon exposure of PBMCs to homopolymers of the isu peptide and to recombinant gp41, the isu domain should be defined as the biologically active domain in gp41 with regards to immunosuppression. This is supported by the fact that mutations in the isu domain of recombinant gp41 abrogated the immunosuppressive activity. However, as mutations might have distal effect acting on an active domain located in another part of gp41 means that the results obtained using the isu mutants are not sufficient to definitively demonstrate an active role for the isu domain. The fact that a peptide corresponding to the isu domain has the same biological activity as recombinant or viral gp41 does, however, strongly suggest that this is indeed the case.

Here a systematic analysis of the influence of the isu domain of gp41 of HIV-1 on cytokine release using cytokine arrays and on gene expression in human PBMCs using a microarray and confirmative real-time PCR was performed. Significant changes in cytokine expression were observed which correlated well with the expression of cytokines in HIV-1 infected individual.

## Materials and Methods

### Peptides, Peptide Conjugates and Polymers

Synthetic peptides, either 17-mers or 19-mers, corresponding to the immunosuppressive domain of gp41 of HIV-1 (HXB2, aa574-592 or aa576-592, access. nr. K03455), (KQ)LQARILAVERYLKDQQL (Figure 1), containing a free carboxyl group at the C-terminus as well as C-terminal amidated peptides and a randomised peptide QRLIQVAEYRLAKQQLLDK were purchased from Genaxxon BioScience GmbH (Biberach, Germany) or from JPT, Jerinin (Berlin, Germany) or were synthesized as described [9]. Peptides were coupled to bovine serum albumin (BSA) using 1-ethyl-3-(3-dimethylamonopropyl) carbodiimide hydrochloride (EDC, Pierce, Rockford, USA) as described [9] or homopolymers of the peptides were produced by cross-linking with EDC and Sulfo-NHS (Pierce) as recommended by the supplier.

### Isolation of Human PBMCs

Donor PBMCs were isolated from whole blood of healthy donors by Ficoll-Hypaque (PAA Laboratories, Austria) density centrifugation using Leucosep tubes (Greiner, Germany). 3×10^5^ cells/well were cultivated with and without peptide homopolymers at 37°C in RPMI 1640 with 10% fetal calf serum (FCS, Biochrome AG, Berlin, Germany) which had been selected for very low induction of IL-10 in normal PBMCs.

### Proliferation Assays

Proliferation assays were performed by stimulating donor PBMCs with 80 µg/ml phytohemagglutinin (PHA, Remel) or 10 µg/ml Concanavalin A (ConA, Sigma) in the presence or absence of peptide conjugates. After addition of ^3^H-thymidine (1 µCi/well) on day zero, one, two or three, the cells were incubated for additional 24 h at 37°C and then harvested. Counts per minute (cpm) were determined with an Inotech automated filter counting system.

### Enzyme-linked Immunosorbent Assays for IL-6, IL-10, sTREM-1 and MMP-1

Supernatants from PMBCs (3×10^5^ cells/well) either untreated or treated for 2–24 hrs with the peptide polymers were collected by centrifugation at 2000 g for 10 min and tested by ELISA. The ELISAs were performed according to the protocols of the suppliers of the kits: IL-6, IL-10 – BD Biosciences, San Diego, USA; sTREM-1 – R&D Systems, Minneapolis, USA, MMP-1 – Raybiotech, Inc., Norcross, USA.

### Cytokine Arrays

Cytokine release from treated or untreated donor PBMCs were measured by membrane-based cytokine arrays I and VI (RayBiotech, Inc., maps 1.1. and 6.1.) after 24 hrs. In the case cells were stimulated with 10 µg/ml Concanavalin A (ConA, Sigma), supernatants were analysed after 3 days. Kinetic studies were performed using supernatant from donor PBMCs, incubated for 6, 8, 16 or 24 hours with isu homopolymer and analysed by the Quantibody™ Human Inflammation Array 1 (RayBiotech). In addition a Multiplex Human Cytokine/Chemokine Magnetic Bead Panel (Millipore) was used to measure cytokine and chemokine release.

### RNA Isolation from PBMCs

Total RNA was isolated from donor PBMCs (3×10^5^/well) using the RNeasy kit (Qiagen, Germany). The RNA concentration was measured using a NanoDrop spectrometer ND-100 (PEQLAB), and RNA specimens were used immediately or kept at −80°C before use.

### Microarray

Total RNA was prepared as described above. An RNA integrity number (RIN) of 9.1 and 9.7 were determined for the RNA from PBMCs cultured with medium or isu-peptide homopolymers, respectively (RIN 10 is the highest). The microarray was performed by IMGM Laboratories, Munich. 0.5 µg of total RNA were converted into digoxigenin (DIG)-labeled cRNA in a RT-IVT reaction, 10 µg were fragmented and hybridised using a Human Genome Survey Microarray V2.0 plate from Applied Biosystems. After washing, an anti-DIG-AP-conjugate (Roche, Germany) was applied and signals were detected with an AB1700 Microarray Reader.

### One-step Real-time Quantitative PCR

One-step real-time RT-PCRs were established for human IL-6, IL-10, MMP-1, TREM-1, FCN-1, CXCL-9 and SEPP-1 (Supplementary Table S1, S2) and duplex PCRs were performed using GAPDH for normalisation (ΔC_t_ = C_t_ gene of interest – C_t_ GAPDH). Total RNA was isolated as described above. Primers and probes for PCR were selected using the Sigma Genosys Probe Design Program and obtained from this company. PCRs were performed in triplicates. Reporter fluorescence was measured using an Mx4000 Multiplex Quantitative PCR System (Stratagene) and evaluated using the 2^−ΔΔCT^ method [22].

### Determination of Single Nucleotide Polymorphism (SNP)

To determine the SNP in the promotor regions of IL-10 and IL-6, DNA was isolated from PBMCs of the donors, and using specific primers (Supplementary Table S3) the corresponding sequences were amplified, sequenced and classified.

### Statistical Analysis

The p values were calculated using the unpaired Student’s t-test.

### Ethical Statement

The use of human blood has been approved by the ethical commission at the Medical Faculty of the Humboldt University Berlin. Written informed consent was provided by study participants.

## Results

### Synthetic Peptides Corresponding to the Isu Domain of gp41 of HIV-1 Inhibit Activation of PBMCs and Modulate Expression of Cytokines

Since peptides corresponding to the isu domain of HIV-1 were biologically active only when conjugated to a carrier [7], [9], we applied the isu peptide of HIV-1 conjugated to bovine serum albumin (BSA) and in parallel we used homopolymers of the isu peptide. Conjugates and polymers were produced by EDC (1-ethyl-3-(3-dimethylamonopropyl) carbodiimide hydrochloride) treatment inducing random covalent COOH-NH_2_ links of defined amino acids (NH_2_ groups in 574K, 575Q, 577Q, 579R, 584R, 587K, 589Q, 590Q, COOH groups in 583E and in the C-terminal 591L, Figure 1a). Both, the isu peptide BSA conjugates and the isu peptide homopolymers, inhibited the activation of PBMCs from healthy human blood donors stimulated with phytohemagglutinin (PHA), a T cell mitogen, in a dose-dependent manner as measured in a proliferation assay (Figure 1c) and induced an increased release of IL-6 and IL-10 (Figure 1d). As control, homopolymers of a peptide with the same sequence but with an amidated C-terminal amino acid were used, which did not induce IL-10 release (Figure 1d), indicating that the C-terminal COOH-group plays a crucial role in the polymerisation process leading to an immunosuppressive polymer. The amount of released IL-10 did depend on the dose of the isu peptide polymer (Figure 1d). When PBMCs of more than 50 donors were treated with the isu polymer or with medium, in all cases an increased release of IL-6 and IL-10 was observed (Supplementary Figure S2a, b). The difference in the IL-10 and IL-6 release by PBMCs treated with the isu peptide homopolymer and by untreated PBMCs was in all cases significant (Supplementary Figure S2a, b, c, d). The IL-10 inducing activity of different batches of isu peptide homopolymer differed slightly (Supplementary Figure S2a, c,d). In addition, the amount of released IL-10 was donor-dependent. When PBMCs from 7 donors were incubated with one and the same batch of the isu peptide homopolymer (Figure 1e) and 9 donors with another (Supplementary Figure S2b), this result was confirmed, indicating that genetic factors of the host were involved. Donor 1 released nearly 700 pg/ml (p = 0.001), donor 7 only 350 pg/ml (p = 0.03) (Figure 1e). The dependence of the release of IL-10 and IL-6 on the donor is stable over time, giving the same results when the release of both cytokines was re-tested 28 and 107 days later (Supplementary Figure S3, S4).

**Figure 2:**
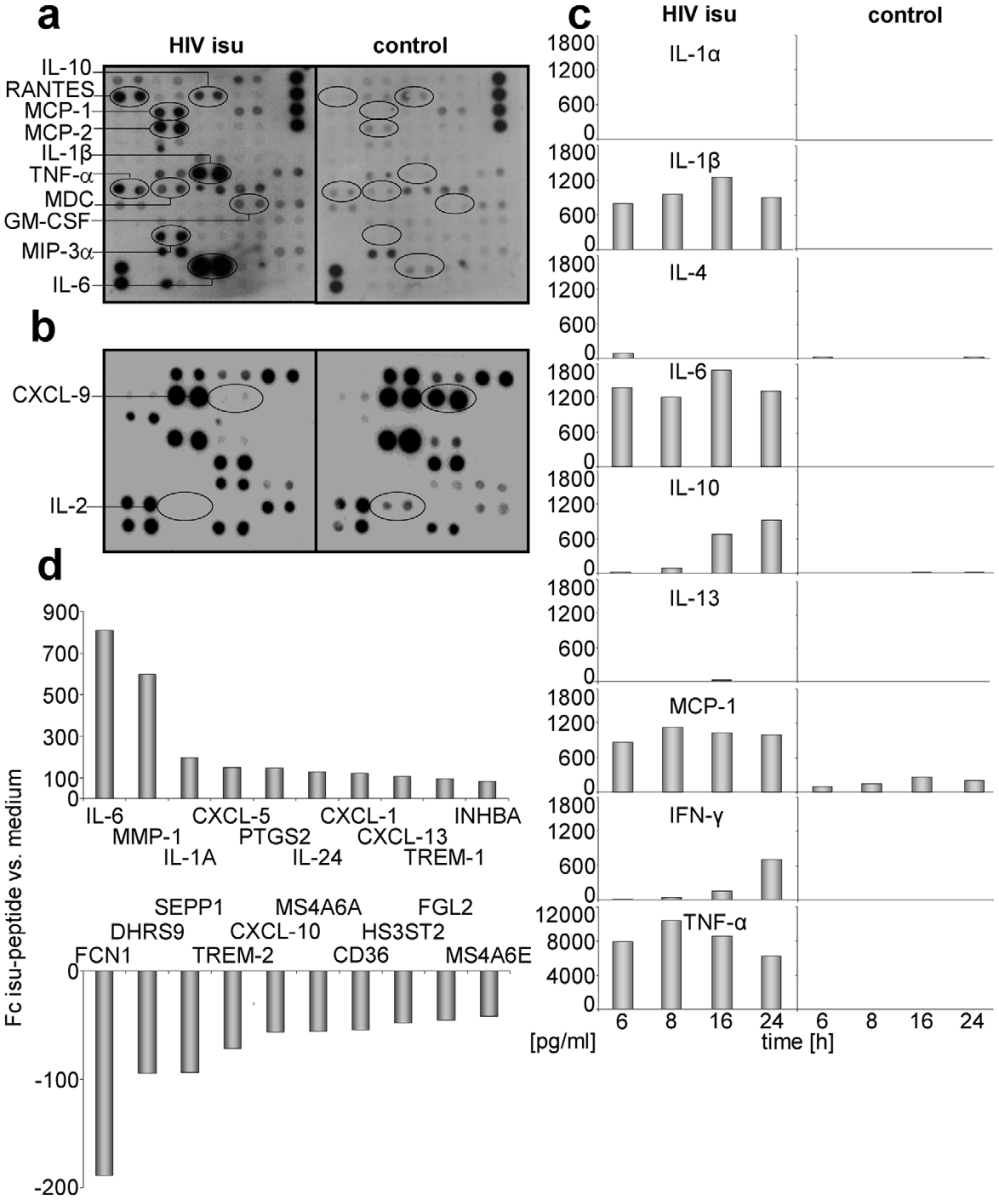
Influence of the isu peptide on cytokine release and gene expression. (a) PBMCs were incubated for 24 hrs with or without the isu peptide homopolymer. The cytokine array VI (see Material and Methods) was used. The up-regulated cytokines are circled. (b) PBMCs were treated with Con A and incubated with and without the isu peptide homopolymer for three days and the cytokine array I was used. The down-regulated cytokines are circled. Similar results were obtained with PBMCs from ten other donors. (c) Cytokine array measuring simultaneous release of ten different cytokines after incubation of PBMCs from one donor with and without the isu peptide homopolymer at different time points (6 to 24 hrs), confirming and extenting the results shown in Figure 2a. Control PBMCs were incubated with medium. Compare the increase in IL-10 expression with that in Figure 3a. (d) Genes with the highest up-regulation (upper part) and down-regulation (lower part) of expression in PBMC of one donor in response to the isu peptide treatment. Using specific real-time PCRs for the up- and down-regulated genes, the changes were confirmed in PBMCs of other donors (Figure 3, 4, Supplementary Figure S2). Fold changes (Fc) indicates gene expression compared to control cells incubated in medium. The full names of the genes are given in Supplementary Table S4.

Four different methods were used to analyse the release of 66 cytokines from PBMCs after incubation with the isu peptide homopolymer. First, membrane based cytokine arrays were used to analyse the cytokine content in the supernatant of PBMCs 24 hrs after exposure to the isu peptide polymer (Figure 2a). An increase in protein expression of IL-6, IL-10, IL-1beta, MCP-1, MCP-2, MIP-3alpha and others was observed. In addition, cytokine release from PBMCs stimulated with Concanavalin A (ConA), another T cell mitogen, and treated simultaneously with the isu peptide homopolymer was measured after 3 days (Figure 2b). This setting allowed to measure decreasing expression of IL-2 and CXCL9. Second, changes in cytokine release over time were studied measuring the cytokine content in the supernatant 6, 8, 12 and 24 hrs after incubation (Figure 2c). In the case of IL-10 and IFN gamma an increase of release was observed, the expression of other cytokines was high (e.g., MCP-1) or low over the whole time. Clear differences were observed when compared with the untreated PBMC cultures (with exception of IL-1 alpha and IL-13 which also had a low expression after incubation of PBMCs with the isu peptide polymer). Third, a multiplex assay based on magnetic beads (not shown) and fourth, confirmative ELISAs specific for each single cytokine were performed (Figure 1d and below).

To summarise, 16 cytokines were found up-regulated, 2 cytokines (CXCL9 and IL-2) were found down-regulated and 48 remained unchanged under these experimental conditions (Table 1, Supplementary Table S4), indicating for the first time that the isu domain modulates the expression of a wide range of human cytokines and chemokines.

**Table 1:** Summary of the changes in cytokine expression.

Up-regulated	IL-1beta, IL-6, IL-8, IL-10, IL-13, GM-CSF, MCP-1 MCP -2, MDC, MIP-1alpha, MIP-1beta, MIP-3alpha, RANTES (CCL5), TNF alpha, IFN gamma, Gro
Down-regulated	IL-2, MIG (CXCL9)
Unchanged	ANG, BDNF, BLC, BMP-4, BMP-6, CKbeta 8-1, CNTF, EGF, Eotaxin, Eotaxin-2, Eotaxin-3, FGF-6, FGF-7, Flt-3 ligand, FKN, GCP-2, GCSF, GDNF, Gro alpha, I-309, IGFBP-1, IGFBP-2, IGFBP-4, IGF-1, IL-1 alpha, IL-1ralpha, IL-3, IL-4, IL-5, IL-7, IL-8, IL-15, IL-16, Leptin, LIGHT, MCP-3, MCP-4, M-CSF, MIP-1delta, NAP-2, NT-3, PARC, PDGF-BB, SCF, SDF-1, TARC, TGF-beta1, TGF-beta3, TNF beta

Cytokine release was measured 24 hrs or 3 days after incubation of normal PBMCs with isu-peptide homopolymers alone or in the presence of a mitogen, respectively. The full name of the abbreviated molecules is given in Supplementary Table S4.

### Synthetic Peptides Corresponding to the Isu Domain of gp41 Modulate Gene Expression

In order to study the influence of the isu peptide on gene expression in human PBMCs, a genome wide microarray analysis was performed using RNA isolated from the PBMCs of a healthy blood donor 24 hrs after incubation with the isu peptide polymer in medium. For comparison RNA of PBMCs incubated with medium alone was used. In this analysis 384 genes were found up-regulated and 360 genes were found down-regulated upon the incubation with the isu peptide. The top ten genes of each group with the highest fold change (up or down) are shown in Figure 2d, the top 50 genes with the highest change in expression are shown in Supplementary tables S5 (up) and S6 (down). The highest up-regulation was shown for the IL-6 gene (Figure 2d, position 1), confirming the results on the protein level (Figure 1d, 2a, c). The genes with higher expression are predominantly involved in processes belonging to “Immunity and defense” and “Signal transduction”.

Among the genes up-regulated at the mRNA level were MMP-1, coding for matrix metalloproteinase 1, a zinc-dependent protease essential for the breakdown of extracellular matrix expressed on monocytes and macrophages [23] (Figure 2d, position 2), and TREM-1 (triggering receptor expressed on myeloid cells 1) (Figure 2d, position 9). TREM-1 has a role as a regulator of innate and adaptive immunity [24], [25]. Among the down-regulated genes were FCN1 (ficolin, position 1 in Figure 2d), CXCL-9/MIG (monokine induced by IFNγ) (position 21, not shown in Figure 2d, position 25 in Supplementary Table S6) and SEPP1 (selenoprotein P, plasma 1, position 3 in Figure 2d). CXCL9 is a chemokine, binding like CXCL10 und CXCL11 to the common receptor CXCR3. FCN1, CXCL9 and SEPP1 play an important role in innate immune responses [26]–[28]. CXCL9 had also been found down-regulated at the protein level in one of the cytokine arrays (Figure 2b).

### Kinetic Studies on Cytokine Release and Gene Expression

**Figure 3:**
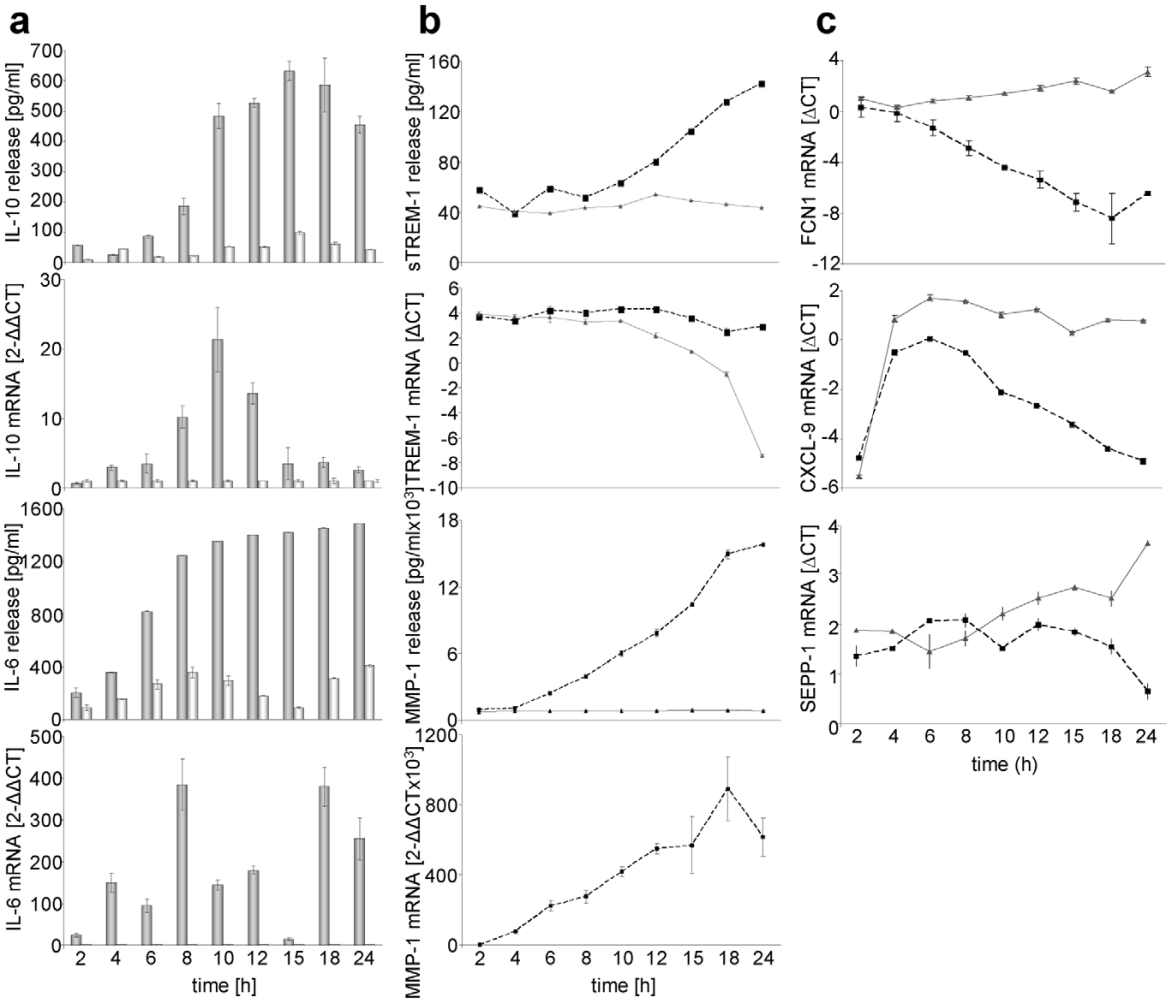
Kinetics of the modulation of cytokine release and gene expression induced by the isu peptide. a, Kinetics of the IL-10 and IL-6 release and expression of IL-10 and IL-6 mRNA. PBMCs were incubated with (gray) or without (light gray) isu peptide homopolymers and supernatants as well as mRNA were collected at different time points between 0–24 hours. The p values were calculated using the Student’s t-test, n = 3. When comparing the IL-10 release induced by the isu peptide homopolymer and that by medium alone, the p value at the peak release (15 hrs) is p = 2.16E-05, the p value for IL-10 RNA at 24 hrs is p = 0.09. All other values were accordingly. b, c, Kinetics of the expression of MMP-1, TREM-1, FCN1, CXCL9 and SEPP-1 in PBMCs incubated with (dotted line) or without (straight line) isu-peptide homopolymers. The figures show a representative result obtained with PBMCs from more than five donors. The p values were calculated using the Student’s t-test, n = 3, the p-value of sTREM-1 release at 24 hrs is p = 0.02.

Surprisingly, no increase in IL-10 mRNA was found in the RNA microarray assay, although expression of IL-10 protein as measured by ELISA (Figure 1d, Supplementary Figure S2) and cytokine array (Figure 2a, c) was significantly increased at the same time (24 hrs) of incubation. To examine this discrepancy, IL-10 mRNA was measured at different time points using a duplex real-time PCR. The level of IL-10 mRNA increased over a period of 10 hrs and decreased nearly to zero at 24 hrs (Figure 3a), thus confirming the low and unchanged expression found in the microarray assay (24 hrs). When we studied the kinetics of IL-6 expression (position 1 among the up-regulated genes in the RNA microarray, Figure 2d), we found that the release of IL-6 protein into the supernatant increased steadily, whereas the expression of IL-6 mRNA showed two peaks of expression at 8 and at 18 hrs (Figure 3a).

Expression of TREM-1 mRNA in cells treated with the isu peptide homopolymer remained unchanged at a high level, whereas the amount of TREM-specific mRNA in the control cells in medium decreased (Figure 3b). This explains the elevated expression of TREM-1 seen in the microarray assay after 24 hrs (Figure 2d). At the same time, using an ELISA, an increase of the amount of a soluble form of TREM-1 without membrane spanning and cytoplasmic domains, sTREM-1, was found in the supernatant of cells treated with the isu peptide homopolymer in comparison with untreated PBMCs (Figure 3b). sTREM is either a splice variant of TREM-1 or, more likely, it is shedded from the plasma membrane [29].

The expression of FCN1 mRNA decreased over time in PBMCs upon incubation with the isu peptide polymer, whereas the expression in control PBMCs did not change significantly (Figure 3c). The expression of the mRNA of CXCL9 decreased in the PBMCs treated with the isu peptide polymer and stayed at a high level in PBMCs in the control medium. The expression of the SEPP1 mRNA decreased in the cells of the culture treated with isu peptide polymer (Figure 3c).

**Figure 4:**
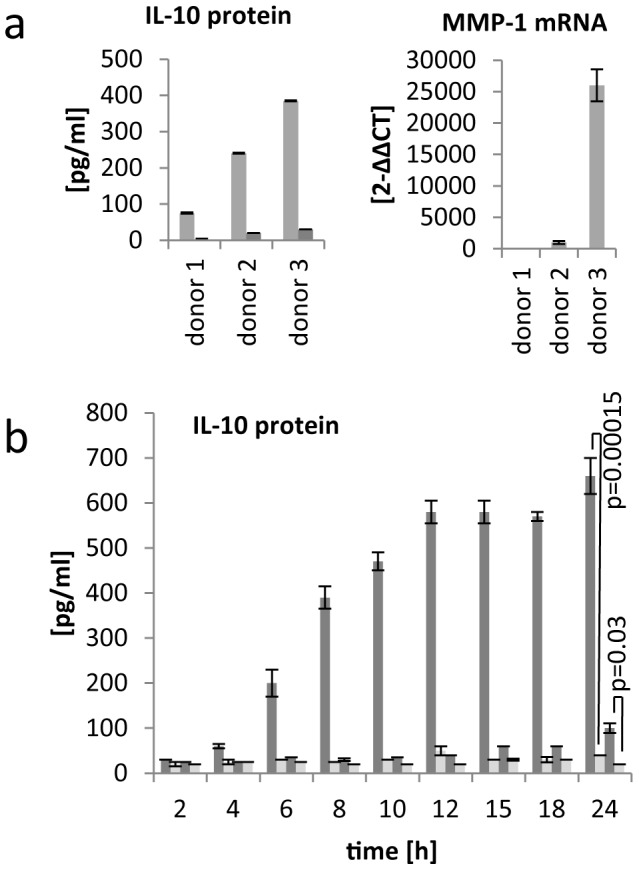
Donor-dependence of IL-10 and MMP-1 release. a, PBMCs from three donors were incubated with the same batch of the isu peptide polymer or medium and IL-10 release and MMP-1 mRNA expression were measured simultaneously. The donor-dependence of IL-10 release was shown using PBMCs from more than 50 donors some are shown in Supplementary Figure S2. b, Kinetic date of IL-10 release from PBMCs from a high responder donor A (column 1, dark grey) and low responder donor B (column 3, dark grey), both treated with the isu peptide homopopymer. Untreated medium control cells from both donors (Colum 2 and 4, light grey) did not release IL-10. The p values were calculated using the Student’s t-test, n = 3, p = 0.00015 in the case of a high responder and p = 0.03 in the case of a low responder.

The release of IL-10 and IL-6 upon incubation of PBMCs with the isu peptide polymers was found to be donor-dependent (more than 100 donors were tested for IL-10 and 12 donors for IL-6, examples were shown in Figure 1e, Supplementary Figure S2). When comparing the expression of IL-10 in PBMCs from seven blood donors, treated with one and the same batch of the isu peptide homopolymer, PBMCs from some donors reacted with a high IL-10 release, others with a lower (e.g., donor 1∶690 pg/ml IL-10, donor 3∶383 pg/ml; Figure 1e, 4a, Supplementary figures 3 and 4). At the same time a donor-dependent difference was also found for other cytokines such as TREM-1 and MMP-1, both measured at the mRNA level (Figure 4a). The decrease of TREM-1 expression was stronger in PBMCs from donor 3 compared with donor 1 (not shown) and the increase of MMP-1 expression was highest in PBMCs of donor 3 compared with that of donor 1 (Figure 4a).

Kinetic studies showed that the differences in IL-10 expression between the donors were not due to a time shift of the peak of release. After incubation of the same batch of the isu peptide homopolymer with PBMCs from two different blood donors, the IL-10 protein release increased over a period of 24 hours in the case of donor 3, whereas the release in the case of donor 1 was much lower at all time-points (Figure 4b). Furthermore, measuring IL-10 release after incubation with the isu peptide polymer four weeks later showed a nearly identical result (634 pg/ml and 626 pg/ml). Therefore in these experiments blood donors reacting with a high and others with a low release of cytokines including IL-10 in response to the isu peptide homopolymer were identified (high and low responders).

To summarise, the changes in the expression of different genes detected in an RNA microarray were confirmed using real-time PCR specific for each gene and in different donors.

### Absence of a Correlation between IL-10 and IL-6 Release and Single Nucleotide Polymorphisms in their Promoter Regions

Since single nucleotide polymorphisms (SNP) in the promoter regions of IL-10 and IL-6 are well studied [30]–[32], we investigated whether the donor-dependent expression of both cytokines (Supplementary Figure S2, Figure 1e) correlated with the SNP in the promoter sequence. We sequenced the IL-10 promoter regions from the DNA of seven donors and in parallel IL-10 release by their PBMCs was measured upon incubation with the same batch of the isu peptide homopolymer. No correlation between the expression of IL-10 and the described SNP was observed. Repeating the measurement of the IL-10 release by PBMCs from these seven donors three times on day 1, day 28 and day 107 confirmed the results (Supplementary Figure S3). This experiment also showed that the amount of the released IL-10 was similar for each donor over time. Only one donor, C, suffering from a common cold on day 28 produced only 20% of the amount released on day 1 or 107 (Supplementary Figure S3).

Similarly, no correlation between the SNP in the IL-6 promoter and the release of IL-6 upon the interaction with the isu peptide polymer was observed (Supplementary Figure S4). Interestingly these data also show that high expression of IL-10 does not automatically correlate with high expression of IL-6.

## Discussion

The isu domain of gp41 of HIV-1 was found to have a broad influence on gene expression and cytokine release by human PBMCs. Since IL-10 and IL-6 showed the highest increase in release among the 16 up-regulated cytokines (Figure 1, 2), we focused on these cytokines. IL-6 showed the highest expression at the mRNA level after 24 hrs (result of the microarray, Supplementary Table S5), whereas IL-10 mRNA showed a peak at 10 hrs and nearly no expression at 24 hrs (Figure 3a). Furthermore, expression of cytokines such as IL-1beta, GM-CSF, MCP-1, MCP-2, MDC, MIP-3alpha, RANTES (CCRL5), and TNF-alpha and of other genes such as MMP-1 was found elevated by the isu peptide polymers. On the other hand, expression of some genes was found down-regulated, among them CXCL-9, FCN1 and SEPP1, which play an important role in innate immunity.

Noteworthy, a similar modulation of cytokine release as shown *in vitro* upon incubation of PBMCs from healthy donors with the isu peptide polymer was found when PBMCs were incubated with gp41 produced in human cells or purified HIV-1 particles [21] as well as in HIV-1 infected individuals [33]–[35]. Therefore these changes may be partially explained by the interaction of the isu domain of gp41 with the immune system. Especially the amount of IL-10 and IL-6 were found significantly increased in HIV-1 infected individuals. Among the cytokines up-regulated in our experiments, also TFN-alpha and IFNgamma were up-regulated in HIV-1 infected individuals [34], [35]. In SIV-infected rhesus macaques, IL-10 production in lymph nodes is already detected at day 7 and increases further by day 28 post-infection [36].

Why the changes in the expression of cytokines and other genes shown in Table 1 and Supplementary tables S5 and S6 will lead to immunodeficiency? First of all, down-regulation of FCN1, CXCL9 and SEPP1 may prevent early local innate immune responses against the virus allowing infection and replication. This is supported by the fact that no HIV-1 sequences with mutations in the isu domain abrogating the immunosuppressive activity were found in patients [21]. Later IL-10 and other cytokines will be induced. IL-10 is a strong immunosuppressive cytokine which is also used by herpes viruses. Some of the induced cytokines may interact with immune cells triggering the expression of MMP-1 and TREM-1 (Figure 3b). MMP-1 may contribute by cutting surface TREM-1 into soluble TREM-1. TREM-1 was shown to induce IL-8, MCP-1 and TNF-alpha and this was also observed in the experiments with the isu peptide (Figure 2a). The increasing amount of replicating virus and the high concentration of IL-10 will inhibit the immune system further, allowing further virus replication, further increase in gp41 and further increase in IL-10 amplifying the immunosuppression.

We were surprised to detect that a matrix metalloproteinase, MMP-1, was up-regulated by the isu peptide polymer. However, MMP-1 expression has been reported to be increased following HIV-1 infection of monocytes/macrophages with cell free virus [37] and expression of MMP-1 at the mRNA and protein level was found increased in infected brain tissues in patients with HIV-1 associated dementia [38]. Like for many other MMPs, expression of MMP-1 is usually low in normal resting tissues and it is transcriptionally regulated by growth factors, hormones and cytokines [39]. Cytokine inducers of MMP-1 include IL-1, -4, -5, -6, -8, -10, and TGF-alpha [39], [40]. As shown in Table 1 the expression of some of them was elevated in our experiments with the isu peptide, so it is likely that changes in cytokine expression increase the expression of MMP-1 also in the HIV-1 infected individual. In this context the function of the MMP-9 is of great interest. MMP-9 expressed on the tumour cell surface cleaves the IL-2 receptors of approaching cytotoxic lymphocytes, so preventing killing of the tumour cell [40]. MMPs have been shown to shed TREM-1 from stimulated human monocytes [41]. The increased expression of MMP-1 in our experiment correlated with the increased shedding of sTREM-1 (Figure 3), suggesting that the induced IL-10 increased the expression of MMP-1 and that increased the release of sTREM.

TREM-1 expression was found on monocytes and neutrophils and upon cross-linking (the ligand is still unknown) TREM-1 induces IL-8 secretion in neutrophils and abundant release of IL-8, TNF-alpha, and MCP-1 in monocytes [42]. An increased expression of sTREM-1, IL-8, TNF-alpha and MCP-1 was also observed after incubation of PBMCs with the isu peptide polymers (Figure 2a, c, 3b). In addition to IL-10 and IL-6, elevated levels of TNF-alpha were also found in HIV-1 infected individuals [34], [42].

The monocyte chemoattractant protein-1(MCP-1/CCL2) is also up-regulated in HIV-1 infected individuals; its plasma level correlates with virus (gp41) load in HIV-1 infection and expression level were diminished after antiviral therapy (for review see [43]). Monocyte-derived macrophages infected with HIV-1 are known to produce TNF-α, IL-1, IL-6, RANTES, MIP-1α, and MIP-1β [44], expression of all these cytokines was also increased upon incubation of normal PBMCs with the isu peptide polymer (Table 1).

Several genes involved in innate immunity such as FCN1, CXCL9 and SEPP1 were found down-regulated by the isu peptide homopolymer. FCN1 has been shown to be involved in the clearance of dying host cells and cellular debris, it is primarily expressed by monocytes, granulocytes and myeloid progenitor cells in the bone marrow but also in the spleen and lung, it is secreted and circulating in the plasma [45]. Down-regulation of FCN1 as shown in our experiments may be useful for the virus *in vivo*, preventing clearance of virus particles. SEPP1, which was also down-regulated in our experiments, is important for the selenium metabolism. 55% of selenium (Se) in the human serum is bound by SEPP1 and people infected with HIV-1 have been reported to be deficient in selenium [46], [47]. A deficiency in Se was strongly associated with decreased survival in HIV-1 disease and application of Se was reported to have a beneficial effect in the treatment of HIV-1 [48], [49]. Daily Se supplementation was shown to suppress the progression of HIV-1 and provide indirect improvement of CD4 count [50]. Taken together, this is the first report showing down-regulation of cytokines involved in innate immunity by the gp41-derived isu domain, suggesting that HIV-1 may use this domain in the very early phase of virus infection to inhibit innate immunity.

In contrast to IL-10 which showed a single peak of mRNA expression at 10 hrs after incubation of human PBMCs with isu peptide polymers, the amount of IL-6 mRNA increased before and after this peak (Figure 3a). When the monocytic cell line THP-1 was incubated with HIV particles or recombinant gp41, release of Il-6 and IL-10 was observed and addition of recombinant IL-10 inhibited IL-6 release [51]. Our and these results suggest an autoregulatory mechanism of cytokine expression.

Changes in cytokine release had been also described for the isu peptide of gammaretroviruses, designated CKS-17 [11], [52]. It was shown that CKS-17 inhibits mitogen-triggered activation of PBMCs and increases IL-10 release [6], [8]. The same was observed for the TM protein of the human endogenous retrovirus HERV-K (our unpublished data). It is important to note here, that in a microarray study, using PBMCs from the one donor, the homopolymers of the isu peptide of HIV-1 (Figure 2d), and the TM protein of HERV-K (unpublished data) induced a nearly identical modulation of the gene expression. Since the isu peptide homopolymer of HIV-1 consists of synthetic peptides and the TM protein of HERV-K was produced in yeast cells, a common contamination in each preparation can be excluded.

The mechanism of the immunosuppressive activity of the isu domain and the corresponding signal transduction is still unclear. In preliminary experiments we showed that - in contrast to the measles virus [53] - the Akt kinase seems not to be involved and siRNA specific for IL-10 reduced the expression of IL-10 but also of other cytokines such as MMP-1, suggesting a key role of IL-10 (unpublished).

The conformation of the isu domain seems to be critical. Soluble peptides were inactive, but isu peptide-BSA conjugates [7], [9] and homopolymeres of isu peptides (Figure 2 and 3) were effective. Since a peptide containing the same amino acid sequence as the isu peptide but containing an amidated C-terminus (-CONH_2_) was ineffective in inducing IL-10 and other cytokines (Figure 2), the involvement of the C-terminal -COOH group in the interaction between -NH_2_ and -COOH groups during polymerisation seems to be crucial. The probability to produce during polymerisation the right conformation able to induce cytokine modulation in the target cell seems to be low. This explains the high amount of polymers which has to be added in order to induce IL-10 or other cytokines. In our parallel study using recombinant gp41 released from transfected human cells or virus released from infected human cells, a 700 fold lower concentration of gp41 was needed to induce the same amount of IL-10 [21]. Most importantly, single mutations in the isu domain of gp41 abrogated the release of IL-10 [21].

The mechanism of interaction between the isu peptide polymers and the target cell is still unknown. The isu peptide polymers might interact with proteins on the cell surface (receptors) as suggested previously [54]–[56]. We recently reported that certain mutations in the isu domain were crucial to abrogate cytokine release whereas others were donor-dependent [21] suggesting a polymorphic receptor.

Viruses developed numerous, often multiple, mechanisms allowing suppression of the innate and adaptive immunity in order to infect the host successfully. There are two principal ways to induce immunosuppression. The first approach uses analogues of cellular cytokines or decoys of cellular receptors, as shown for herpes and pox viruses [57], [58]. The second approach is based on a direct interaction of viral proteins with the immune system as shown for the measles [59], [60] and the Ebola virus [61]. Retroviruses including HIV-1 seem to belong to this group using their TM protein to contribute to the immunodeficiency.

What are the implications of this finding? In the infected individual gp41 will be found in virus particles after shedding of the surface envelope protein gp120, in immune complexes with antibodies reactive against gp41 as well as on the surface of infected cells. The largest amount of gp41 will certainly be found on the surface of cells in the lymph nodes and other lymphoid organs where gp41 may interact directly with neighbouring uninfected cells and contribute significantly to the immunosuppression in HIV-1 infected individuals inhibiting the innate and adaptive immunity. This gp41 induced immunosuppression may promote replication of the virus and decrease immune protection against opportunistic infections. In the course of infection and progression to AIDS the immunosuppressive effect will increase as a consequence of the increase of virus/gp41 load. The clinical picture of AIDS is composed of the immune responses against HIV-1 and to other microorganisms, leading to an activation of the immune system, and simultaneously a severe immunosuppression, which when untreated, is fatal. The TM protein gp41 of HIV-1 contribute to this severe immunosuppression. Since we had shown that antibodies against the immunosuppressive domain inhibited the immunosuppressive effect [10], such antibodies or antibodies against the putative receptor or specific inhibitors may be used as antiviral agents.

## Supporting Information

Figure S1 **Evolutionary conservation of the sequence of the isu domain.** Human and simian immunodeficiency viruses were analysed, (.) marks identical amino acids, (*) marks deletions. Hatched amino acids represent conservative exchanges (A = S = T, I = L = M = V, R = K).
(TIF)

Figure S2 **Dependence of the Il-10 and IL-6 release on the donor PBMCs and the isu peptide polymer.** A, PBMCs from one donor (A) was treated with 25 µg each of 3 different isu homopolymers (HP1-HP3), b, PBMCs from 9 donors (B to J) were treated with one and the same batch of an isu peptide homopolymer (HP4), c, PBMCs from one donor (L) was treated with 5 different isu peptide homopolymers (HP5-HP9) d, PBMCs from one donor (M) was treated with 3 different isu peptide homopolymer (HP10– HP12). The cytokine release by PBMCs after treatment with the isu peptide homopolymer was compared with the cytokine release after the treatment with medium alone and the p values were determined using the Student′s t test. The IL-10 release by PBMCs treated with medium alone was nearly zero in all cases.
(TIF)

Figure S3 **Absence of a correlation between the IL-10 release by PBMCs from 7 different donors and their SNP in the IL-10 promotor.** The PBMCs were incubated with the isu peptide homopolymer for 24 hrs at three different time points, the sequence of the relevant promoter region of each donor was determined and genetically expected IL-10 production is indicated. Donor B was not available at day 107, donor C had a cold on day 28.
(TIF)

Figure S4 **Absence of a correlation between the IL-6 release by PBMCs from 7 different donors and their SNP in the IL-10 promotor.** Their PBMCs were incubated with the isu peptide homopolymer for 24 hrs at two different time point, the promoter of each donor was sequenced and the genetically expected IL-6 release is indicated. Donor B was not available at day 107.
(TIF)

Table S1 **Primers for the real-time RT-PCR analysis.**
(DOC)

Table S2 **Probes for the real-time RT-PCR analysis.**
(DOC)

Table S3 **Primers used for sequencing.**
(DOC)

Table S4 **Abbreviation and full names of the cytokines studied in the microarrays (** **Table 1** **).**
(DOCX)

Table S5 **Fifty cytokines with the highest increase in expression** **upon incubation of PBMCs with isu peptide polymers.** The order is the result of a microarray comparing RNA from PBMCs incubated with the isu peptide homopolymer and from PBMCs incubated with medium. The expression of 27,000 genes was analysed.
(DOCX)

Table S6 **Fifty cytokines with the highest reduction in expression upon incubation of PBMCs with isu peptide polymers.** The order is the result of a microarray comparing RNA from PBMCs incubated with the isu peptide homopolymer and from PBMCs incubated with medium. The expression of 27,000 genes was analysed.
(DOCX)
