# Supplementary figures and images for: Modulation of Cytokine Release and Gene Expression by the Immunosuppressive Domain of gp41 of HIV-1
Source: PLoS One. 2013 Jan 30;8(1):e55199. doi: 10.1371/journal.pone.0055199 (PMC3559347; doi:10.1371/journal.pone.0055199)

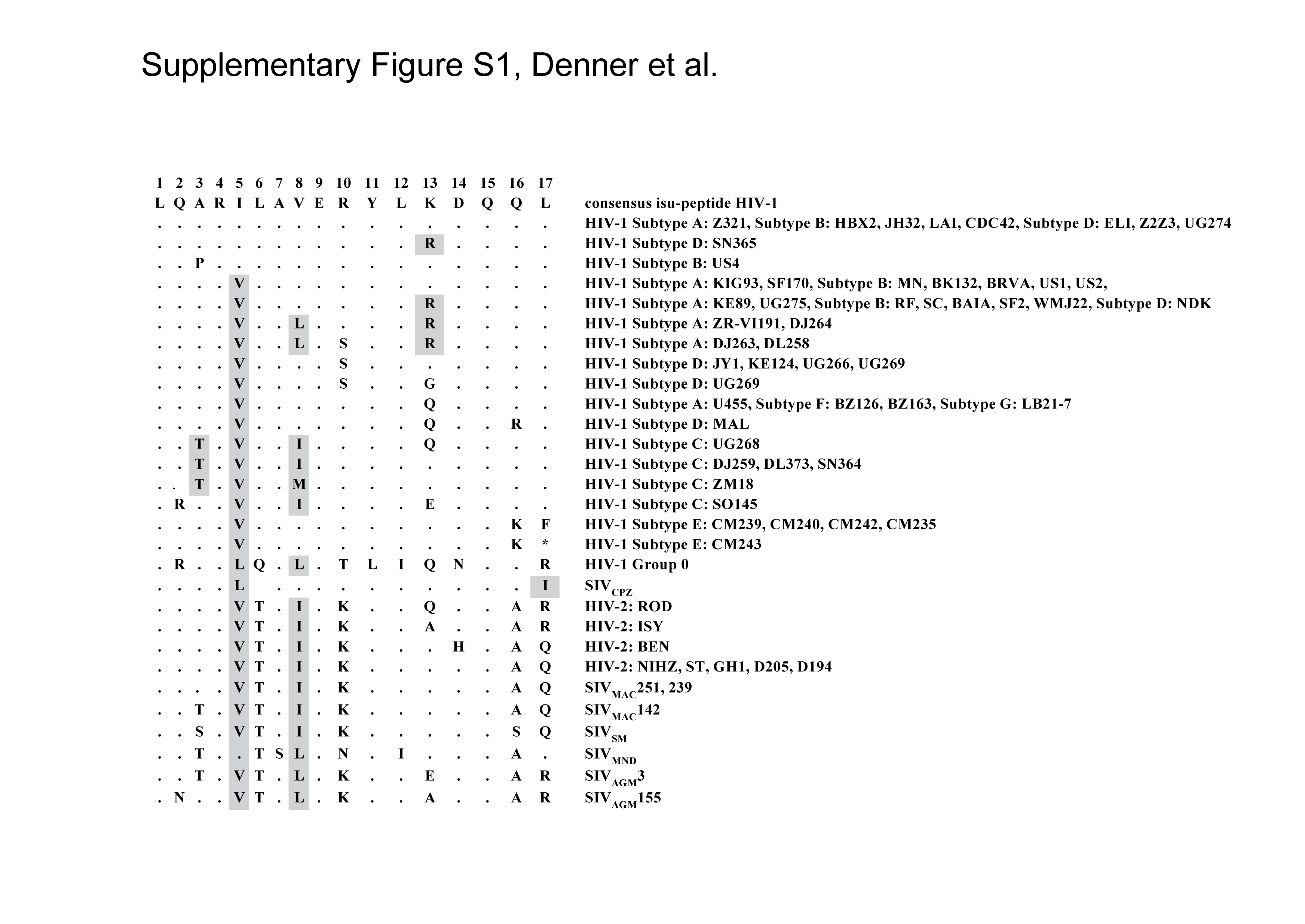

Supplement: Figure S1 — Evolutionary conservation of the sequence of the isu domain. Human and simian immunodeficiency viruses were analysed, (.) marks identical amino acids, (*) marks deletions. Hatched amino acids represent conservative exchanges (A = S = T, I = L = M = V, R = K). (TIF) [file pone.0055199.s001.tif]

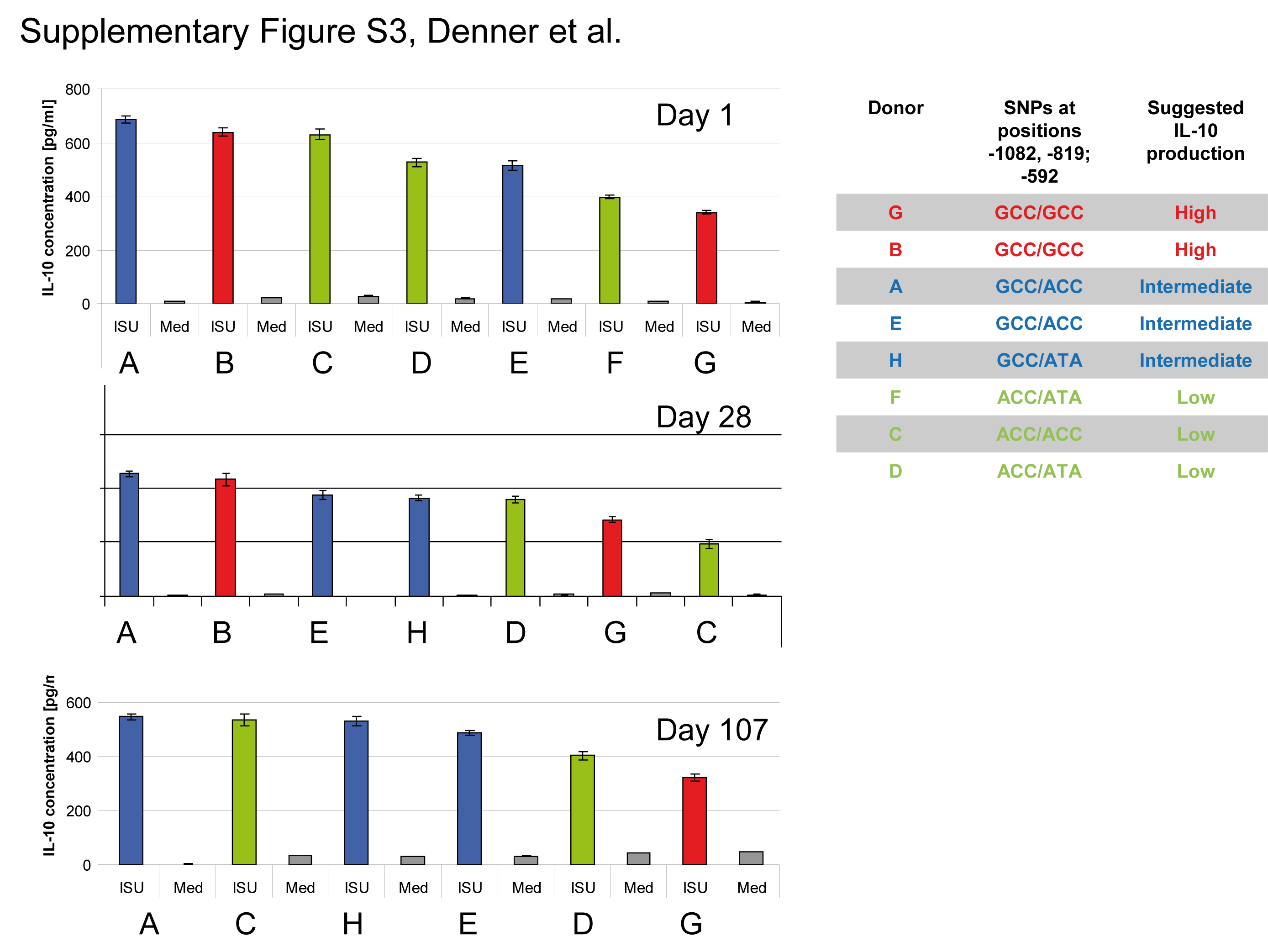

Supplement: Figure S3 — Absence of a correlation between the IL-10 release by PBMCs from 7 different donors and their SNP in the IL-10 promotor. The PBMCs were incubated with the isu peptide homopolymer for 24 hrs at three different time points, the sequence of the relevant promoter region of each donor was determined and genetically expected IL-10 production is indicated. Donor B was not available at day 107, donor C had a cold on day 28. (TIF) [file pone.0055199.s003.tif]

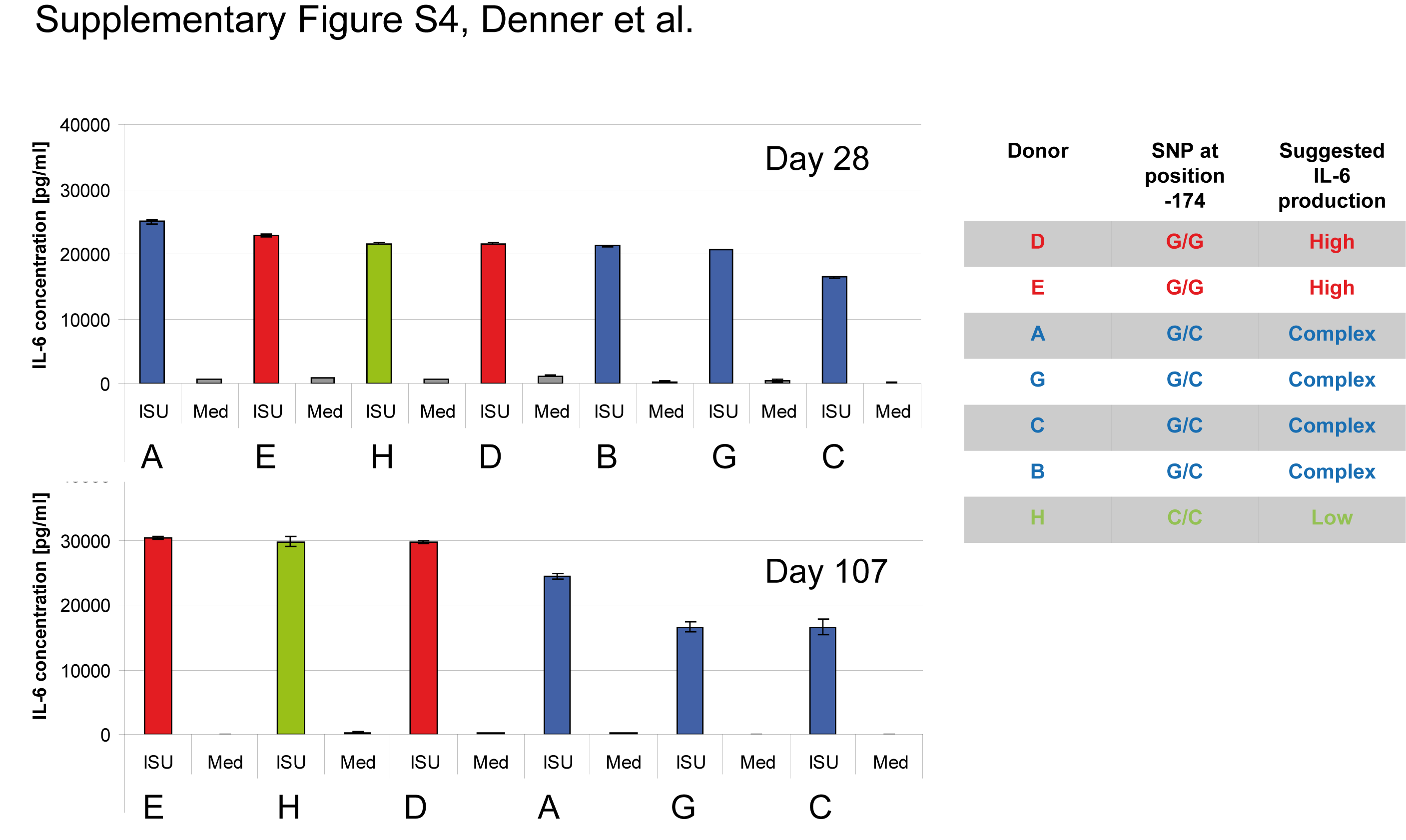

Supplement: Figure S4 — Absence of a correlation between the IL-6 release by PBMCs from 7 different donors and their SNP in the IL-10 promotor. Their PBMCs were incubated with the isu peptide homopolymer for 24 hrs at two different time point, the promoter of each donor was sequenced and the genetically expected IL-6 release is indicated. Donor B was not available at day 107. (TIF) [file pone.0055199.s004.tif]
