# Supplementary material for: Modulation of Cytokine Release and Gene Expression by the Immunosuppressive Domain of gp41 of HIV-1
Source: PLoS One. 2013 Jan 30;8(1):e55199. doi: 10.1371/journal.pone.0055199 (PMC3559347; doi:10.1371/journal.pone.0055199)
Supplement: Table S1 — Primers for the real-time RT-PCR analysis. (DOC) [file pone.0055199.s005.doc]

**Supplementary Table S1**. Primers for real-time RT-PCR analyses

| **Gene** | **Accession nr** | **Sequence** | **nt - nt** |
| --- | --- | --- | --- |
| hsGAPDH | NM 002046.3 | 5´GGCGATGCTGGCGCTGAGTA3´ | 364-386 |
|  |  | 3´TGGTTCACACCCATGACGA5´ | 494-512 |
| hsIL-10 | NM 000572.2 | 5´CCACGCTTTCTAGCTGTT3´ | 966-983 |
|  |  | 3´CTCCCTGGTTTCTCTTCCTAA5´ | 1058-1078 |
| hsIL-6 | NM 000600.3 | 5´GGTACATCCTCGACGGCATCT3´ | 289-309 |
|  |  | 3´GTGCCTCTTTGCTGCTTTCAC5´ | 349-369 |
| hsMMP-1 | NM 002421.3 | 5´CATCCAAGCCATATATGGACG3´ | 908-928 |
|  |  | 3´TCTGGAGAGTCAAAATTCTCT5´ | 1498-1518 |
| hsTREM-1 | NM 018643.2 | 5´GCCTCACATGCTGTTCGAT3´ | 427-445 |
|  |  | 3´GGTACAAATGACCTCAGCGT5´ | 746-765 |
| hsFCN1 | NM 002003.3 | 5’CTGCAAGGACCTGCTAGACC3’ | 440-459 |
|  |  | 5’CTACCTACCGAGACACCTGA3’ | 572-591 |
| hsCXCL9 | NM_002416 | 5’GGAGTGCAAGGAACCCCAGTA3’ | 94-114 |
|  |  | 3’TCTTTCAAGGATTGTAGGTGGATAGTC5’ | 151-178 |
| hsSEPP1 | NM 005410.2 | 5’CATGGACATCAGCACCTT3’ | 774-459 |
|  |  | 3’TCGACAGAGCTTCTTTTG5’ | 954-972 |
