# Supplementary material for: Modulation of Cytokine Release and Gene Expression by the Immunosuppressive Domain of gp41 of HIV-1
Source: PLoS One. 2013 Jan 30;8(1):e55199. doi: 10.1371/journal.pone.0055199 (PMC3559347; doi:10.1371/journal.pone.0055199)
Supplement: Table S2 — Probes for the real-time RT-PCR analysis. (DOC) [file pone.0055199.s006.doc]

**Supplementary Table S2**. Probes for real-time RT-PCR analyses

| **Gene** | **Accession nr** | **Sequence** | **nt - nt** |
| --- | --- | --- | --- |
| hsGAPDH | NM 002046.3 | Hex-CTTCACCACCATGGAGAAGGCTGGG[3BQ1] | 405-429 |
| hsIL-10 | NM 000572.2 | 6Fam-TCTTGTCTCTGGGCTT[3BQ1] | 1015-1030 |
| hsIL-6 | NM 000600.3 | 6Fam-TGTTACTCTTGTTACATGTCTCCTTTCTCAGGGCT[3BQ1] | 311-345 |
| hsMMP-1 | NM 002421.3 | 6Fam-CTGGGCTGTTCAGGGACAGAA[3BQ1] | 1187-1207 |
| hsTREM-1 | NM 018643.2 | 6Fam-CAGCCAGGAGAATGACAATGTTGA[3BQ1] | 675-698 |
| hsFCN1 | NM 002003.3 | 6Fam-TATTTCCTGAGCGGCTGGCACA[BHQ1] | 465-486 |
| hsCXCL9 | NM002416 | 6Fam-TCGCTGTTCCTGCATCAGCACCA[BHQ1] | 126-148 |
| hsSEPP1 | NM 005410.2 | 6Fam-AGAATCAGCAACCAGGAGCA[BHQ1] | 721-740 |
