# Supplementary material for: Modulation of Cytokine Release and Gene Expression by the Immunosuppressive Domain of gp41 of HIV-1
Source: PLoS One. 2013 Jan 30;8(1):e55199. doi: 10.1371/journal.pone.0055199 (PMC3559347; doi:10.1371/journal.pone.0055199)
Supplement: Table S3 — Primers used for sequencing. (DOC) [file pone.0055199.s007.doc]

**Supplementary Table S3**. Primers used for sequencing

| **Gene** | **Accession nr** | **Sequence** | **length of the amplicon** |
| --- | --- | --- | --- |
| hsIL-10 | NM 000572 | 5´ TTCCCCAGGTAGAGCAACAC3´ | 860 |
| hsIL-10 | NM 000572 | 5´CCTAGGTCACAGTGACGTGG3´ |  |
| hsIL-6 | NM 000600.3 | 5´GCCACGCGGTGGCAAAAAGG3´ | 659 |
| hsIL-6 | NM 000600.3 | 5´TTTCGTTCCCGGTGGGCTCG3´ |  |
