# Supplementary material for: Modulation of Cytokine Release and Gene Expression by the Immunosuppressive Domain of gp41 of HIV-1
Source: PLoS One. 2013 Jan 30;8(1):e55199. doi: 10.1371/journal.pone.0055199 (PMC3559347; doi:10.1371/journal.pone.0055199)
Supplement: Table S4 — Abbreviation and full names of the cytokines studied in the microarrays ( Table 1 ). (DOCX) [file pone.0055199.s008.docx]

**Supplementary Table S4**. Abbrevations and full names of the cytokines shown in Figure 2a. The cytokines with elevated expression are in bold, with reduced expression in italic (see Figure 2a).

| Abbreviation | | Full name | |
| --- | --- | --- | --- |
| ANG | Angiogenin | |  |
| BDNF | Brain-derived Neurotrophic Factor | |  |
| BLC | B-lymphocyte Chemoattractant | |  |
| BMP-4 | Bone Morphogenetic Proteins -4 | |  |
| CK β 8-1 | Chemokine beta-8 | |  |
| CNTF | Ciliary Neuronotrophic Factor | |  |
| EGF | Epidermal growth factor | |  |
| Eotaxin | CCL11 | |  |
| Eotaxin-2 | MPIF-2 (Myeloid Progenitor Inhibitory Factor 2 ), CCL24 | |  |
| Eotaxin-3 | MIP-4-alpha (macrophage inflammatory protein-4-alpha), CCL26 | |  |
| FGF-6 | Fibroblast Growth Factor-6 | |  |
| FGF-7 | Fibroblast Growth Factor-7 | |  |
| Fit-3 Ligand | fms-like Tyrosine Kinase-3 Ligand | |  |
| FKN | Fractalkine | |  |
| GCP-2 | Granulocyte Chemotactic Protein 2 | |  |
| GCSF | Granulocyte Colony-Stimulating Factor | |  |
| GDNF | Glial-derived Neurotrophic Factor | |  |
| GM-CSF | **Granulocyte macrophage colony-stimulating factor** | |  |
| GRO | Growth Related Oncogene | |  |
| GRO alpha | Growth Related Oncogene-Alpha | |  |
| I-309 |  | |  |
| IFN-gamma | Interferon-gamma | |  |
| IGFBP-1 | Insulin-like Growth Factor Binding Proteins 1 | |  |
| IGFBP-2 | Insulin-like Growth Factor Binding Proteins 2 | |  |
| IGFBP-4 | Insulin-like Growth Factor Binding Proteins 4 | |  |
| IGF-I | Insulin-like growth factor-1 | |  |
| IL-10 | **Interleukin-10** | |  |
| IL-13 | Interleukin-13 | |  |
| IL-15 | Interleukin-15 | |  |
| IL-16 | Interleukin-16 | |  |
| IL-1alpha | Interleukin-1 alpha | |  |
| IL-1beta | **Interleukin-1 beta** | |  |
| IL-1ralpha | Interleukin-1 receptor alpha | |  |
| *IL-2* | *Interleukin-2* | |  |
| IL-3 | Interleukin-3 | |  |
| IL-4 | Interleukin-4 | |  |
| IL-5 | Interleukin-5 | |  |
| IL-6 | Interleukin-6 | |  |
| IL-7 | Interleukin-7 | |  |
| IL-8 | Interleukin-8 | |  |
| lep. | Leptin | |  |
| LIGHT | an acronym derived from: homologous to **l**ymphotoxins, **i**nducible expression, competes  with HSV **g**lycoprotein D for **H**VEM, a receptor expressed on **T**-lymphocytes | |  |
| MCP-1 | **Monocyte Chemoattractant Protein 1** | |  |
| MCP-2 | **Monocyte Chemoattractant Protein 2** | |  |
| MCP-3 | Monocyte Chemoattractant Protein 3 | |  |
| MCP-4 | Monocyte Chemoattractant Protein 4 | |  |
| M-CSF | macrophage colony stimulating factor; monocyte colony stimulating factor | |  |
| MDC | Macrophage-derived Chemokine | |  |
| *MIG (CXCL9)* | *Monokine induced by Gamma Interferon* | |  |
| MIP-1alpha | Macrophage Inflammatory Protein 1 Alpha | |  |
| MIP-1bata | Macrophage Inflammatory Protein 1 Beta | |  |
| MIP-1delta | Macrophage Inflammatory Protein 1 Delta | |  |
| MIP-3alpha | Macrophage Inflammatory Protein 3 alpha | |  |
| NAP-2 | Neutrophil Activating Peptide 2 | |  |
| NT-3 | Neurotrophin-3 | |  |
| PARC | Pulmonary and Activation-Regulated Chemokine | |  |
| PDGF-BB | Platelet-derived Growth Factor BB | |  |
| RANTES | **Regulated upon activation T-cell expressed and presumably secreted** | |  |
| SCF | Stem Call Factor | |  |
| SDF-1 | Stromal Cell-derived Factor | |  |
| TARC | Thymus and Activation-Regulated Chemokine | |  |
| TGF-beta 1 | Tumor Necrosis Factor beta-1 | |  |
| TGF-beta 3 | Tumor Necrosis Factor beta-3 | |  |
| TNF-alpha | **Tumor necrosis factor-alpha** | |  |
| TNF-beta | Tumor necrosis factor-beta | |  |
