# Supplementary material for: Modulation of Cytokine Release and Gene Expression by the Immunosuppressive Domain of gp41 of HIV-1
Source: PLoS One. 2013 Jan 30;8(1):e55199. doi: 10.1371/journal.pone.0055199 (PMC3559347; doi:10.1371/journal.pone.0055199)
Supplement: Table S5 — Fifty cytokines with the highest increase in expression upon incubation of PBMCs with isu peptide polymers. The order is the result of a microarray comparing RNA from PBMCs incubated with the isu peptide homopolymer and from PBMCs incubated with medium. The expression of 27,000 genes was analysed. (DOCX) [file pone.0055199.s009.docx]

**Supplementary Table S5**. Fifty cytokines with the highest increase in expression upon incubation of PBMCs with isu peptide polymers

| Abbreviation | Full name |
| --- | --- |
| IL6 | interleukin 6 (interferon, beta 2) |
| MMP1 | matrix metallopeptidase 1 (interstitial collagenase) |
| IL1alpha | interleukin 1, alpha |
| CXCL5 | chemokine (C-X-C motif) ligand 5 |
| PTGS2 | prostaglandin-endoperoxide synthase 2 (prostaglandin G/H synthase and cyclooxygenase) |
| IL24 | interleukin 24 |
| CXCL1 | chemokine (C-X-C motif) ligand 1 (melanoma growth stimulating activity, alpha) |
| CXCL13 | chemokine (C-X-C motif) ligand 13 (B-cell chemoattractant) |
| TREM1 | triggering receptor expressed on myeloid cells 1 |
| INHBA | inhibin, beta A (activin A, activin AB alpha polypeptide) |
| IL1beta | interleukin 1, beta |
| SGNE1 | secretory granule, neuroendocrine protein 1 (7B2 protein) |
| IL1F9 | interleukin 1 family, member 9 |
| CA12 | carbonic anhydrase XII |
| KIAA1295 | KIAA1295 |
| ARNT2 | aryl-hydrocarbon receptor nuclear translocator 2 |
| CCL20 | chemokine (C-C motif) ligand 20 |
| CXCL3 | chemokine (C-X-C motif) ligand 3 |
| EREG | epiregulin |
| IL8 | interleukin 8 |
| CXCL6 | chemokine (C-X-C motif) ligand 6 (granulocyte chemotactic protein 2) |
| PI3 | peptidase inhibitor 3, skin-derived (SKALP) |
| MMP12 | matrix metallopeptidase 12 (macrophage elastase) |
| PTGES | prostaglandin E synthase |
| TM4SF1 | transmembrane 4 L six family member 1 |
| INSM1 | insulinoma-associated 1 |
| TFPI2 | tissue factor pathway inhibitor 2 |
| SERPINB7 | serpin peptidase inhibitor, clade B (ovalbumin), member 7 |
| CCL14\|CCL15 | chemokine (C-C motif) ligand 14\|chemokine (C-C motif) ligand 15 |
| HAS1 | hyaluronan synthase 1 |
| C1QTNF1 | C1q and tumor necrosis factor related protein 1 |
| THBD | thrombomodulin |
| AQP9 | aquaporin 9 |
| KIAA0460 | KIAA0460 |
| SPRR2F | small proline-rich protein 2F |
| TNIP3 | TNFAIP3 interacting protein 3 |
| DNER |  |
| MCEMP1 |  |
| NDP | Norrie disease (pseudoglioma) |
| CCL3L1 | chemokine (C-C motif) ligand 3-like 1 |
| EMR3 | egf-like module containing, mucin-like, hormone receptor-like 3 |
| KRTHB6 | keratin, hair, basic, 6 (monilethrix) |
| CSPG2 | chondroitin sulfate proteoglycan 2 (versican) |
| G0S2 | G0/G1switch 2 |
| F3 | coagulation factor III (thromboplastin, tissue factor) |
| MMP14 | matrix metallopeptidase 14 (membrane-inserted) |
| CCL3 | chemokine (C-C motif) ligand 3 |
| SLC16A10 | solute carrier family 16 (monocarboxylic acid transporters), member 10 |
| RETN | resistin |
