# Supplementary material for: Modulation of Cytokine Release and Gene Expression by the Immunosuppressive Domain of gp41 of HIV-1
Source: PLoS One. 2013 Jan 30;8(1):e55199. doi: 10.1371/journal.pone.0055199 (PMC3559347; doi:10.1371/journal.pone.0055199)
Supplement: Table S6 — Fifty cytokines with the highest reduction in expression upon incubation of PBMCs with isu peptide polymers. The order is the result of a microarray comparing RNA from PBMCs incubated with the isu peptide homopolymer and from PBMCs incubated with medium. The expression of 27,000 genes was analysed. (DOCX) [file pone.0055199.s010.docx]

**Supplementary Table S6**. Fifty cytokines with the highest reduction in expression upon incubation of PBMCs with isu peptide polymers

| Abbreviation | Full name |
| --- | --- |
| FCN1 | ficolin (collagen/fibrinogen domain containing) 1 |
| DHRS9 | dehydrogenase/reductase (SDR family) member 9 |
| SEPP1 | selenoprotein P, plasma, 1 |
| ALDH1A1 | aldehyde dehydrogenase 1 family, member A1 |
| TREM2 | triggering receptor expressed on myeloid cells 2 |
| CXCL10 | chemokine (C-X-C motif) ligand 10 |
| MS4A6A | membrane-spanning 4-domains, subfamily A, member 6A |
| CD36 | CD36 antigen (collagen type I receptor, thrombospondin receptor) |
| HS3ST2 | heparan sulfate (glucosamine) 3-O-sulfotransferase 2 |
| FGL2 | fibrinogen-like 2 |
| CLEC10A | C-type lectin domain family 10, member A |
| MS4A6E | membrane-spanning 4-domains, subfamily A, member 6E |
| GPNMB | glycoprotein (transmembrane) nmb |
| FCN2 | ficolin (collagen/fibrinogen domain containing lectin) 2 (hucolin) |
| DHRS9 | dehydrogenase/reductase (SDR family) member 9 |
| RNASE1 | ribonuclease, RNase A family, 1 (pancreatic) |
| FUCA1 | fucosidase, alpha-L- 1, tissue |
| A2M | alpha-2-macroglobulin |
| GPR34 | G protein-coupled receptor 34 |
| CAMP | cathelicidin antimicrobial peptide |
| FLJ22662 | 0 |
| APOC1 | apolipoprotein C-I |
| CPVL | carboxypeptidase, vitellogenic-like |
| TSPAN4 | tetraspanin 4 |
| CXCL9 | chemokine (C-X-C motif) ligand 9 |
| EPHB2 | EPH receptor B2 |
| ASGR1 | asialoglycoprotein receptor 1 |
| FABP3 | fatty acid binding protein 3, muscle and heart (mammary-derived growth inhibitor) |
| KCNJ5 | potassium inwardly-rectifying channel, subfamily J, member 5 |
| PMFBP1 | polyamine modulated factor 1 binding protein 1 |
| VSIG4 | V-set and immunoglobulin domain containing 4 |
| ASRGL1 | asparaginase like 1 |
| ADORA3 | adenosine A3 receptor |
| MNDA | myeloid cell nuclear differentiation antigen |
| CD302 | CD302 antigen |
| TLR7 | toll-like receptor 7 |
| OSBPL1A | oxysterol binding protein-like 1A |
| ADORA3 | adenosine A3 receptor |
| SERPING1 | serpin peptidase inhibitor, clade G (C1 inhibitor), member 1, (angioedema, hereditary) |
| SIRPB1 | signal-regulatory protein beta 1 |
| CD163L1 | CD163 antigen-like 1 |
| LGMN | legumain |
| CEBPA | CCAAT/enhancer binding protein (C/EBP), alpha |
| FOLR2 | folate receptor 2 (fetal) |
| PTGFRN | prostaglandin F2 receptor negative regulator |
| SDC3 | syndecan 3 (N-syndecan) |
| ACP5 | acid phosphatase 5, tartrate resistant |
| NLN | neurolysin (metallopeptidase M3 family) |
| IGSF2 | immunoglobulin superfamily, member 2 |
| RNASE6 | ribonuclease, RNase A family, k6 |
